# Repeated vs. Acute Exposure of RAW264.7 Mouse Macrophages to Silica Nanoparticles: A Bioaccumulation and Functional Change Study

**DOI:** 10.3390/nano10020215

**Published:** 2020-01-27

**Authors:** Anaëlle Torres, Bastien Dalzon, Véronique Collin-Faure, Thierry Rabilloud

**Affiliations:** Laboratory of Chemistry and Biology of Metals, University Grenoble Alpes, CNRS, CEA IRIG-LCBM, 38000 Grenoble, France; bastien.dalzon@cea.fr (B.D.); veronique.collin@cea.fr (V.C.-F.)

**Keywords:** nanoparticles, macrophage, bioaccumulation

## Abstract

Synthetic amorphous silica is used in various applications such as cosmetics, food, or rubber reinforcement. These broad uses increase human exposure, and thus the potential risk related to their short- and long-term toxicity for both consumers and workers. These potential risks have to be investigated, in a global context of multi-exposure, as encountered in human populations. However, most of the in vitro research on the effects of amorphous silica has been carried out in an acute exposure mode, which is not the most relevant when trying to assess the effects of occupational exposure. As a first step, the effects of repeated exposure of macrophages to silica nanomaterials have been investigated. The experiments have been conducted on in vitro macrophage cell line RAW264.7 (cell line from an Abelson murine leukemia virus-induced tumor), as this cell type is an important target cell in toxicology of particulate materials. The bioaccumulation of nanomaterials and the persistence of their effects have been studied. The experiments carried out include the viability assay and functional tests (phagocytosis, NO and reactive oxygen species dosages, and production of pro- and anti-inflammatory cytokines) using flow cytometry, microscopy and spectrophotometry. Accumulation of silica nanoparticles (SiO_2_ NP) was observed in both exposure scenarii. However, differences in the biological effects between the exposure scenarii have also been observed. For phagocytosis, NO production and Tumor Necrosis Factor (TNF) release, repeated exposure tended to induce fewer effects than acute exposure. Nevertheless, repeated exposure still induces alterations in the macrophage responses and thus represents a scenario to be tested in detail.

## 1. Introduction

Silica nanoparticles SiO_2_NP are used in many applications such as cosmetics, food or rubber reinforcement [1,2]. According to a report of the Ministère de l’Energie et des ressources naturelles of Quebec, based on the U.S. Geological Survey data, the world production of silica was 142 million tons in 2013, making it one of the most produced nanomaterials. The precipitated silica, which is produced by a wet route in a solvent, is used as a rubber reinforcing agent and as cleaning and polishing agent in toothpastes [3]. It is also used as an anti-caking agent in food and pharmaceutical additives [4]. Finally, the food-grade silica, known as E551 food additive agent, is commercially available as amorphous silica, but is not considered as nanosilica [5]. Although its primary particle is a nanoparticle (NP) of ~20 nm in diameter, E551 occurs in clusters of micrometer size, which is similar to the fumed silica but does not contain crystalline domains, and the ingested dose is estimated to 35 mg per day [6].

The crystalline silica is well known to be toxic, and its effects are irreversible. The proinflammatory response is at the origin of the silicosis (a lung chronic inflammation disease) [7,8]. In this context, the effects of the industrial amorphous silica have to be investigated. The silica effects are already well referenced for high doses during an short period [7,9,10]. For example, Joshi et al. have observed the specific phagocytosis phenomenon of silica particles, which results in a phagolysosomal leakage and cell death for both crystalline and amorphous silica. Following the phagocytic activity, reactive oxygen species (ROS) were produced, such as hydrogen peroxide, but in the case of silica, H_2_O_2_ may be converted in a more toxic radicals [11]. Regarding the ROS generation in RAW264.7 cells, an increased level of ROS was observed following an acute exposure [12]. Furthermore, after 24h of silica exposure, the secretion of proinflammatory cytokines was increased as shown by Park and Park and also Di Cristo et al. for two types of silicas (pyrogenic and precipitated) [12,13]. However, the increasing uses of silica, in many daily consumer products, raise the question of the chronic exposure of workers and more widely the general population, by various routes, e.g., inhalation and intestinal. To study the effects of amorphous silica in a context of chronic exposure, the macrophages, i.e., scavenging immune cells that ingest all sorts of particulate material, have been used. The main function of this cell type is the phagocytosis of foreign substances, such as exogenous pathogens. The other functions are the antigen presentation to T cells for the initiation of the specific immune response and the signaling to amplify the immune response with the release of cytokines and nitric oxide.

Moreover, knowing that the two most risky routes for nanosilica exposure are inhalation and intestinal absorption, the macrophages encountered in these two sites have to be studied. First, the intestines are exposed to various elements from the outside and contain a wide bacterial population: part of the gut microbiota. The silica NP could be found in food, the problem of its passage through the intestinal barrier arises. To shed light on this event in the intestinal model, studies on the translocation of particles into and across the gastrointestinal barrier have been realized, especially the transcytosis pathway via the M-cell layer (microfold cells) of the Peyer’s Patches, which also contain macrophages [14,15]. This site represents an important immune sensor with a constant surveillance of pathogens and antigens of the intestinal lumen, containing the largest population of resident macrophages in the body. The second important site is represented by the lungs, which are the center of the air exchange, and makes them a privileged site for the infection originating from inhalation pathway. In the lungs, the immune cells are also present and are mainly localized at the alveolar surface. Alveolar macrophages represent 95% of the bronchoalveolar immune cells. Together with 1 to 4% lymphocytes and 1% neutrophils, they are the sentinel phagocytic cell of the innate immunity [16].

Note that the vast majority of studies on the effects of nanoparticles on cells use an acute exposure scheme in which the cells are exposed to a high but subtoxic concentration of nanoparticles and the effects are read immediately after exposure. Thus, there is a need for studies in which a repeated exposure scenario is used, which is a better mimic of consumer or occupational (and not accidental) exposures. Furthermore, biological responses can be different after acute or repeated exposures, as exemplified in the literature for silver [17], zinc oxide [18], or titanium dioxide [19,20] nanoparticles.

We thus decided to investigate the effects of the exposure modes on macrophages, using fluorescent amorphous nanosilicas. In the present study, we have exposed macrophages to low doses of silica for four days to follow the chronic effect of SiO_2_NP on the cell metabolism, cytoskeleton, and immune function, and compared the results to those obtained by the classical acute exposure scheme.

## 2. Materials and Methods

Most experiments were performed as described in previous publications [21,22]. The details are given here for clarity and readers’ convenience.

### 2.1. Nanoparticles Characterization with Dynamic Light Scattering (DLS)

Sicastar^®^-greenF (FITC) (ref 42.00.301, emission 485 nm, excitation 510 nm) was purchased from Micromod Partikeltechnologie (GmbH, Rostock, Germany). LUDOX^®^ LS colloidal silica was purchased from Sigma-Aldrich (ref 420808) (Merck, Darmstadt, Germany) and is produced by Grace. The size of the nanoparticles was determined after dilution in water or in culture medium by means of dynamic light scattering using a Wyatt Dynapro Nanostar instrument (Wyatt Technology, Santa Barbara, CA, USA). All these silicas are produced by a wet route starting from organosilane precursors [23]. The study has been carried out with the green fluorescent silica because it has been reported that the fluorescein is a stable and nontoxic dye [24]. This polar anionic compound passes poorly through the membrane. Consequently, it can be internalized only through NP internalization and, once internalized, cannot escape from the cytoplasm by leaching, allowing the count of NP internalization.

### 2.2. Bacteria Stimulation

*Escherichia coli* bacteria were grown in agitated liquid cultures at 37 °C in lysogeny broth (LB) medium up to an OD600 of 0.7. The bacteria were harvested by centrifugation, rinsed 3 times in warm (37 °C) phosphate buffer saline (PBS) supplemented with 1 g/L of glucose, and resuspended in 50 mL PBS-glucose containing 16 µg/mL rhodamine B isothiocyanate. The bacteria were maintained under agitation at 37 °C for 30 min in this medium. The bacteria were then collected by centrifugation and rinsed three times in 50 mL PBS. The suspension was sterilized by incubation at 70 °C for 2 h. From the initial OD value of 0.7, the suspension contained 500 million cells/ml, and was diluted in sterile PBS to the adequate concentrations prior to use to obtain a concentration of 10 or 30 bacteria per cell in the plates of macrophage culture.

### 2.3. Cell Culture of RAW264.7 Cells and Viability Assay

RAW264.7 murine macrophage cells were cultured in Roswell Park Memorial Institute (RPMI) (ThermoFisher, Waltham, MA, USA) 1640 medium supplemented with 10% FBS (fetal bovine serum) and 5 μg/mL Ciprofloxacin. Cells were seeded every 2 days at 250,000 cells/mL and harvested at 1 million cells/mL. For repeated exposure (4 days), the cells were seeded into 6-well treated plates at 100,000 cells/mL. After 2 days of culture to reach confluence, the silica dose was added every day and the medium was changed every 2 days.

After silica exposure for 24 h or some days, the viability of the cells was tested. The cells were harvested and flushed with PBS 1X washed with phosphate buffered saline (PBS) and centrifuged for 5 min at 1200 rpm (200 g). Pellets are resuspended in PBS1X-Sytox Blue (final concentration 2 µg/mL, excitation 444 nm, and emission 480 nm; ThermoFisher S34857). Cells were analyzed with a FacsCalibur flow cytometer equipped with the CellQuest software (6.0, Becton Dickinson Biosciences, Le Pont-de-Claix, France) or a FacsMelody flow cytometer (BD Biosciences) equipped with FacsChorus software (1.3, BD Biosciences).

### 2.4. Phagocytosis Activity Measurement

The fluorescent beads have a diameter of 500 nm, which is close to a bacterial size. This assay illustrates the phagocytosis of pathogen by macrophages; the red fluorescence allows quantifying the phagocytic activity of the cell and the green fluorescence corresponding to silica internalization. The cell culture was performed as described previously. Briefly, cells were seeded into 6-well plates and exposed to the indicated dose of silica for twenty-four hours at 37 °C. The red fluorescent latex beads (Sigma L3280, excitation 575 nm, emission 610 nm) were first coated in a FBS 10%—PBS 1X solution for 30 min at 37 °C. This solution was then added to cell culture, and the culture returned to the incubator for 3 h. Then cells were harvested and washed with PBS, centrifuged 5 min at 1200 rpm, the pellets were suspended with 3 mL of water for a few seconds and 1 mL of NaCl (3.5%) was added under vortex mixing to restore the osmotic pressure. The cells were centrifuged and the pellets were resuspended with 200 μL of PBS 1X—Sytox Blue 2 μg/mL, and analyzed by flow cytometry.

### 2.5. Nitric Oxide Production

This experiment is used to determine if there is an interference of silica with the NO production by macrophages induced by bacteria or the bacterial wall component lipopolysaccharide (LPS). LPS induces a strong proinflammatory response, resulting into a detectable NO_2_ production measured as nitrite in the culture medium. Cells grown into 6-well plates were exposed to the indicated dose of silica particles for twenty-four hours at 37 °C and also primed or not with LPS (100 ng/mL) or bacteria for 18 h. L-arginine monohydrochloride (5mM) was also added for 15 h, the supernatants are collected and centrifuged to eliminate nonadherent cells. Then, 500 μL of Griess reagent were added to 500 μL of supernatants, from sample and standards (0 to 50 μM in NO_2_ using a Standard Nitrite solution). The mixture was incubated at room temperature for 20 min, and the absorbance read at 540 nm with a Jenway 7315 spectrophotometer.

### 2.6. Cytokine Dosage in RAW264.7

Cytokines are involved in many homeostatic processes and notably in immune response. This signaling pathway can modulate macrophage functions and cell surface marker expression; they are central actors and markers of the inflammation.

The supernatants were obtained as described above. The experiment was carried out with the Cytometric Bead Array Mouse Inflammation Kit (BD Biosciences), and analyzed with FCAP Array software (3.0, BD Biosciences). This Flex-set kit allows measuring Interleukin-6 (IL-6) and TNF protein levels in a single sample. The mixed capture beads were added to the all assay tubes containing supernatant samples and standards (from 0 to 5000 pg/mL), the mouse inflammation phycoerythrin (PE, excitation 488 nm, emission 575 nm) detection reagent was added, and the mixture was incubated for 2 h at room temperature, protected from light. The wash buffer was added to each tube, which were then centrifuged 5 min at 200 g, the pellets were resuspended with the wash buffer, and analyzed by FacsCalibur flow cytometer.

### 2.7. Immunofluorescence of RAW264.7 Cells in Microscopy

Cells were seeded on glass coverslips at 100,000 cells/mL and exposed to the indicated dose of silica and/or bacteria. Cells were fixed with 4% paraformaldehyde for 30 min and permeabilized with 0.1% Triton-X100 (Eurobio, GAUTTR00-01) for 5 min. Cells were stained with fluorescently labelled phalloidin, which detects polymerized actin (Sigma-Aldrich, Merck, phalloidin-Atto 550) in a final concentration of 500 nM for 20 min at room temperature, protected from light. Nuclei were stained with Vectashield mounting medium containing DAPI (Vector laboratories, Vectashield H-1200). Microscope analysis was performed on a Zeiss LSM 880 microscope (Zeiss, Marly le Roi, France) (confocal). Fluorescence pictures were taken at the same exposure and gain conditions to allow comparison of fluorescence intensity. The raw data were treated and adjusted by using the same parameters with the ImageJ software (1.52s, Wayne Rasband National Institutes of Health, USA).

### 2.8. Numerical Analyses

The Student *t*-test was applied to all the results. The experiments were conducted 2 or 3 times on independent biological replicates. Data are presented as means ± standard deviations; * *p* ≤ 0.05, ** *p* ≤ 0.01, *** *p* ≤ 0.001. Note that the systematic use of propidium iodide or Sytox Blue (ThermoFisher) allows analysis of live cells only.

## 3. Results

### 3.1. Nanoparticle Characterization

Industry sells their product by communicating some characteristics, including a mean particle size in the case of nanomaterials. We had to verify the size of the SiO_2_ NP by using DLS. The three commercial silica nanoparticles were characterized as shown in Table 1.

The dynamic light scattering (DLS) method allows obtaining the hydrodynamic diameter of the particle. The suppliers provide sizes for their particles in distilled water, which were verified. The fluorescent silicas were given for 30 nm in diameter, and in water, the green silica (SiG) showed a diameter of 34 nm. The colloidal silica (LS30) had a diameter of 22 nm. It is however known that the particles can form agglomerates in culture medium, which could modify their internalization by the cells, and consequently their effects [25]. These NPs have been characterized in the medium used (RPMI 10% FBS), and showed some aggregation. The fluorescent NPs were present in two populations 25% have a diameter of 10 nm and 75% a diameter of 50 nm. The LS30 was distributed in 10 nm (20%) and 65 nm (80%) NP diameters, the size distribution diagrams can be found in supplementary data (Table S1). Thus, the NPs tended to form agglomerates but their sizes remained nanoparticular.

### 3.2. Silica Accumulation in Various Exposure Modes

#### 3.2.1. Internalization and Fluorescence Measurement in Acute Exposure

**Figure 1:**
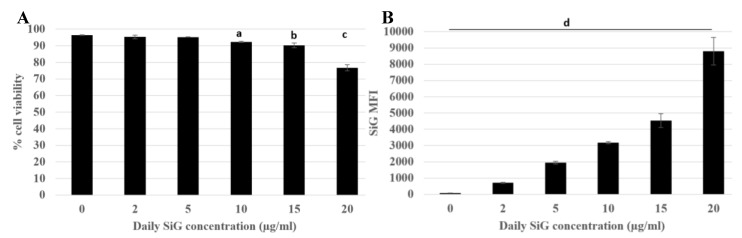
Green silica internalization and viability of RAW264.7 cells after an acute exposure. Green silica MFI obtained with BD Facs Melody flow cytometer. (**A**) Cell viability of exposed cells (a: Control versus acute 10 µg/mL, *p* value < 0.01; b: control versus acute 15 µg/mL, *p*-value < 0.05; c: Control versus acute 20 µg/mL, *p* value < 0.01). (**B**) SiG mean fluorescence intensity depending on NP concentration, (d: all the conditions are significantly different from the control *p*-value < 0.05). The figure represents the duplicate means of three independent experiments, *n* = 6.

First, the effects of silica were tested on the cell lines. The viability assay was carried out with different doses and different silica, with an acute exposure scenario (24 h) to determine the lethal dose 20 (LD20), at which 20% of the cells died and the effects of the treatment were visible. Therefore, for the amorphous LS30 silica and the green fluorescent silica (SiG) this dose corresponded to 20 μg/mL for the RAW264.7 cell line (data not shown). The fluorescent dye did not impact the cell mortality compared with the non-fluorescent silica. The first scenario studied was the acute exposure, which allowed verifying the sensitivity of the flow cytometer. Different doses were tested and the results are presented on Figure 1. The fluorescence intensity increased almost linearly with the concentration of silica as long as the concentration remained lower than the toxic doses. Indeed, the silica green MFI of cells exposed to 5 µg/mL is 1700, the MFI was 3100 for 10 µg/mL exposed cells and for 20 µg/mL exposed cells the MFI was about twice more than for the 10 µg/mL SiG dose with a MFI of 8700. As the background fluorescence of unexposed cells was around 180, this means in then that flow cytometry was sensitive enough to detect the green (SiG) silica fluorescence intensity even at low, non-lethal doses. The obtained values were significantly different from each other (*p*-value lower than 0.05), and this was used to follow the silica NP internalization by the macrophages. The MFI value can change according to the SiG internalization by the cells, but throughout all the experiments, the MFI were always dose-dependent.

#### 3.2.2. Silica Accumulation for a 4-Day Exposure

**Figure 2:**
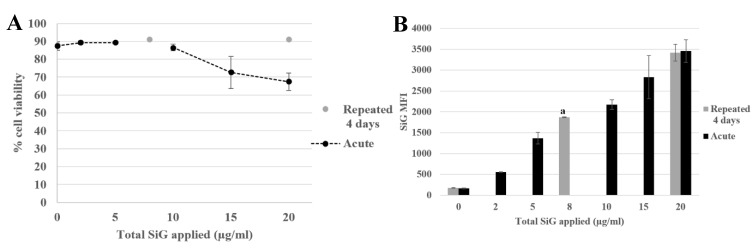
4-day exposure repeated exposure of RAW264.7 cells to SiO_2_ NP compared with acute exposure, by flow cytometer. (**A**) Viability assay of SiG exposed cells measured with propidium iodide. (**B**) Green silica (SiG) internalization. (a: repeated 2 µg/mL versus acute 5 and 10 µg/mL, *p*-value < 0.05).

Herein, the aim was to determine if the daily exposure to silica has a toxic effect on the immune system or not, in the frame of the well-known and irreversible effects of crystalline silica. This chronic exposure mimicked the daily exposure of the workers in industry but also the consumers who absorb food-grade silica in many powdered products. The chronic scenario was tested with a four-day exposure, as illustrated in Figure 2, for the green silica. The viability was measured for the repeatedly exposed cells and for the cells exposed for 24 h. The cells of both scenarios were seeded the same day, which allowed to obtaining the result for an acute exposure on cells with the same ageing in culture.

It appeared that the applied silica doses did not impact cell viability, either in repeated or acute dose, except for the acute 20 µg/mL (LD20). The silica fluorescence was measured in cells. The fluorescence intensity obtained for repeatedly exposed cells (2 μg/mL as a daily dose for 4 days corresponding to 8 μg/mL a total dose) led to a MFI of 1700, i.e., higher than the one obtained for a single exposure to 2 µg/mL silica (500) in the same dose applied in one acute exposure, and corresponded to the MFI intermediate between the one obtained for cells exposed to a single 5 µg/mL dose and the one obtained for cells exposed to a single 10 µg/mL dose. The same phenomenon was observed with the repeated exposure to 5 µg/mL for 4 days compared with the acute dose of 20 µg/mL. In summary, the silica was internalized and accumulated in the cells upon repeated exposure, although the fluorescent obtained after a repeated exposure was always lower than the one observed after a single exposure to the same cumulated dose. This observation was crucial for the study of the amorphous silica toxicity. Indeed, the regulatory agencies have defined an occupational exposure limit corresponding to 10 mg amorphous silica/m^3^ of air and less than 5 mg/m^3^ present in the alveolar fraction. These limitations have taken into account the accumulation of the silica in the organism. A single dose could be nontoxic but the accumulation could lead to some health problems. As a matter of fact, a dose applied at once (acute) or in a cumulative way (repeated) may not lead to the same cellular responses. In this context, the effects of a repeated exposure to amorphous silica have to be seriously investigated, due to the presence of SiO_2_ NP in the industry and in many daily products, which exposes the workers and the consumers to a chronic exposure.

### 3.3. Biological Effect of the Silica Accumulation in Macrophages

#### 3.3.1. Morphological Changes

**Figure 3:**
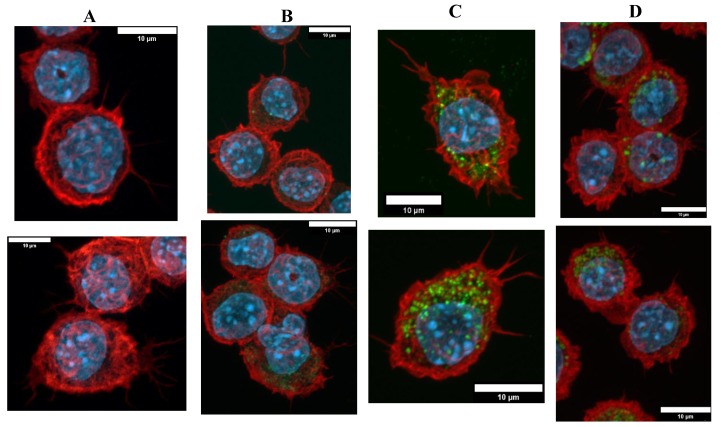
Green silica internalization and cytoskeleton changes of RAW264.7 cells after acute exposure. (**A**–**D**) Control and green silica internalization of cells exposed to 5 µg/mL, 20 µg/mL, and 30 µg/mL during 24 h, respectively, obtained by confocal microscopy (nucleus in blue (DAPI), actin in red (phalloidin), silica in green (FITC)), *n* = 2.

The cells exposed to Sicastar^®^ fluorescent silica NP were observed in fluorescent microscopy to check the NP internalization and to verify the cytoskeleton of the macrophages. The results are presented in Figure 3 with confocal microscopy images of fixed RAW264.7 cells exposed to green fluorescent silica for 24 h. There was heterogeneity for both parameters: the silica NP internalization and the cell cytoskeleton. First, the cell morphologies were diverse with highly adherent cells with long actin filaments and round cells with a few, short filaments. Despite this heterogeneity, there was a tendency in which the silica-exposed cells were less adherent, and it appeared that the cells exposed at the LD20 dose were less spread out than the control, unexposed cells, or cells exposed to lower doses. This phenomenon appeared to be correlated with the applied dose of silica. Regarding the silica internalization, the microscope was less sensitive than the flow cytometers and it did not allow quantifying the SiG fluorescence without specific software.

**Figure 4:**
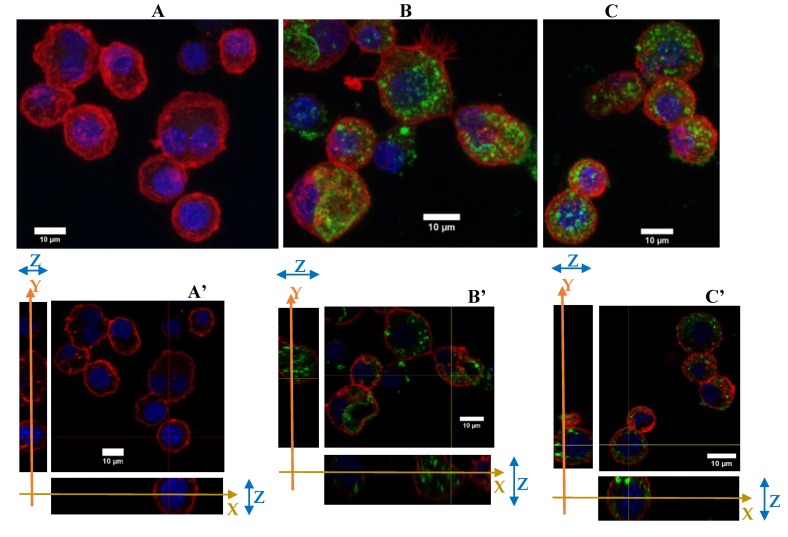
Green silica internalization and cytoskeleton changes of RAW264.7 cells after chronic and acute exposure. (**A**–**C**) Control and green silica internalization of cells exposed repeatedly to 5 µg/mL during 4 days, and 10 µg/mL during 24 h, respectively, obtained by confocal microscopy (nucleus in blue (DAPI), actin in red (phalloidin-atto 550), silica in green (FITC), *n* = 2. Z-project reconstitution. (**A’**–**C’**) Same conditions as previously, represented in orthogonal views. The actin cell membrane is labeled in red by phalloidin-atto550, the nucleus is labeled in blue and the silica nanoparticles are green fluorescent. The stack of the cells corresponding to a slice in the thickness of the cells is illustrated. The combination of the *XY* and *YZ* confocal plans demonstrate the internalization of the silica nanoparticles.

Then, as presented in Figure 4, cytoskeleton changes were observed for repeatedly exposed cells. As a reminder, the cells have been maintained for 4 days at confluence. Under these conditions the cells were less adherent than in the previous figure, where the cells have been maintained for 24 h only. After 4 days of NP exposure, the cells were more vesicular than the acutely exposed cells. Although the silica NP were dispersed in all the cytoplasm, there were more widespread in the repeated exposure scenario.

To conclude with the cytoskeleton, the silica seems to have induced a change in the cytoskeleton of the cells by reducing their filaments and their adherence abilities, for cells cultured for 48 h overall. The NPs were well dispersed in the cytoplasm with an important number of vesicles.

#### 3.3.2. Functional Changes

**Figure 5:**
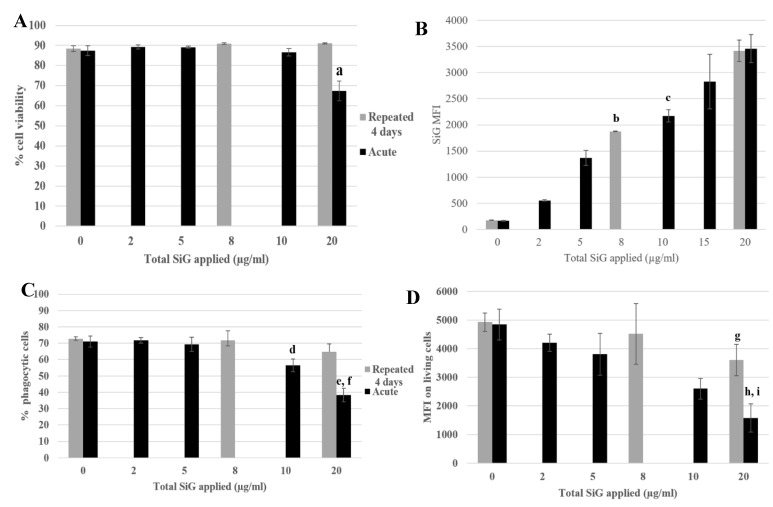
4-day repeated exposure of RAW264.7 to SiG NP compared with acute exposure. (**A**) Cell viability with propidium iodide. (a: CTL versus acute 20 µg/mL, *p*-value < 0.01). (**B**) SiG internalization (b: acute 5 µg/mL versus repeated 2 µg/mL, *p*-value < 0.01; c: acute 10 µg/mL versus repeated 2 µg/mL, *p*-value < 0.05). (**C)** Proportion of phagocytic cells (d: CTL versus acute 10 µg/mL, *p*-value < 0,01; e: CTL versus acute 20 µg/mL, *p*-value < 0.01; f: repeated 5 µg/mL versus acute 20 µg/mL, *p* value < 0.01). (**D**) Phagocytosis of the cells. (g: CTL versus repeated 5 µg/mL, *p*-value < 0,05; h: CTL versus acute 20 µg/mL, *p*-value < 0.01; i: repeated 5 µg/mL versus acute 20 µg/mL, *p* value < 0.05).

To investigate the impact of nanosilica on macrophages, some important functions of the macrophages were tested. The results of the phagocytosis assay are presented in Figure 5.

The cell viability and the silica internalization are presented to confirm the cell exposure to NP (Figure 5A,B) and the red beads uptake is presented in Figure 5C,D, corresponding to the percentage of phagocytic cells and the phagocytic activity of these positive cells. The cells exposed repeatedly to 5 µg/mL (20 µg/mL in a cumulative dose) showed a similar proportion of phagocytic cells as in the control, but with a lower phagocytic activity (a 25% decrease). The cells acutely exposed to 20 µg/mL have a lesser proportion of phagocytic cells compared with the control (decrease of 50%) and also a less phagocyte activity, with a decrease of 70% (Figure 5D). The phagocyte function of acutely exposed cells was thus clearly more impacted than for the repeatedly exposed cells. This was also verified for the repeated 4 × 2 µg/mL-exposed cells compared with the 10 µg/mL acute dose. To conclude about phagocytosis, while acutely exposed cells showed a marked decrease in their phagocytic activity, the decrease was much less important for repeatedly exposed cells. Both the proportion of phagocytic cells and the intensity of phagocytosis, as determined by the MFI of the fluorescent latex beads used for the assay, were higher for repeatedly exposed cells than for acutely exposed ones, although the silica internalization was similar for the two exposure scenarios.

**Figure 6:**
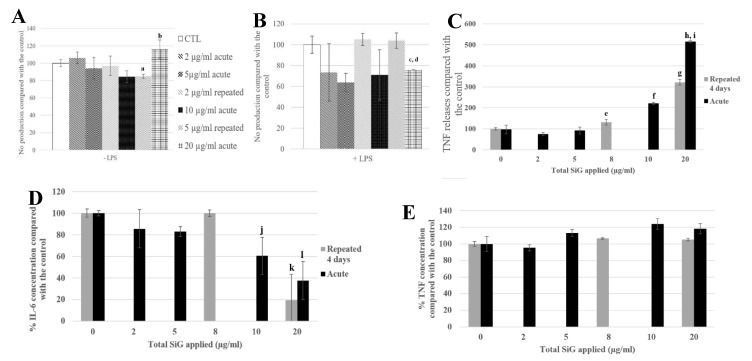
4-day chronic exposure of RAW264.7 to SiG NP and 18 h to LPS compared with silica acute exposure. (**A**,**B**) NO dosage with the Griess reagent by spectrophotometry without or with LPS stimulation (a: CTL versus repeated 5 µg/mL, *p*-value < 0.05; b: repeated 5 µg/mL versus acute 20 µg/ml, *p* value < 0.01, c: CTL versus acute 20 µg/mL, *p*-value < 0.01; d: repeated 5 µg/mL versus acute 20 µg/mL, *p*-value < 0.05). (**C**) TNF releases of RAW264.7 cells exposed to SiG only, measured by flow cytometry (e: CTL versus repeated 2 µg/mL, *p*-value < 0.05; f: CTL versus acute 10 µg/mL, *p*-value < 0.001; g: CTL versus repeated 5 µg/mL, *p*-value < 0.001; h: CTL versus acute 20 µg/mL, *p*-value < 0.001; i: repeated 5 µg/mL versus acute 20 µg/mL, *p*-value < 0.01). (**D**,**E**) Cytokine release measurement of LPS stimulated cells by flow cytometry, respectively IL-6 and TNF. (j: CTL versus acute 10 µg/mL, *p*-value < 0.05; k: CTL versus repeated 5 µg/mL, *p*-value < 0.001; l: CTL versus acute 20 µg/mL, *p*-value < 0.01).

The signaling functions of the macrophages were tested, as shown in Figure 6, with the nitric oxide (NO) production and the cytokine release. The cells were exposed to SiG (acutely or repeatedly), and to lipopolysaccharides (LPS) for 18 h or not, to mimic bacteria encounter. As a reminder, LPS is a component of Gram-negative bacterial membrane. For cells not stimulated with LPS (Figure 6A,C), the 4 × 5 µg/mL-repeatedly exposed cells showed a slight decrease of 15% of the basal NO production compared with the control, whereas the 20 µg/mL-acutely exposed cells produced 16% more NO. Regarding the basal TNF release, the repeatedly-exposed cells produced 3 times more TNF than the control, and the acutely exposed cells 5-fold more than the control. For cells stimulated with LPS, the NO production of the repeatedly exposed cells (4 × 5 µg/mL) was 5% lower than the control, and the acute 20 µg/mL was 30% lower. Concerning IL-6 release, there was a decrease of 80% of the repeated exposed cells (4 × 5 µg/mL) release compared to the control, and a decrease of 70% for cells exposed to a single 20 µg/mL dose. No significant changes were observed in the LPS-induced TNF release.

#### 3.3.3. Physiological Situation of Co-Exposure: Industrial SiO_2_ and LPS

**Figure 7:**
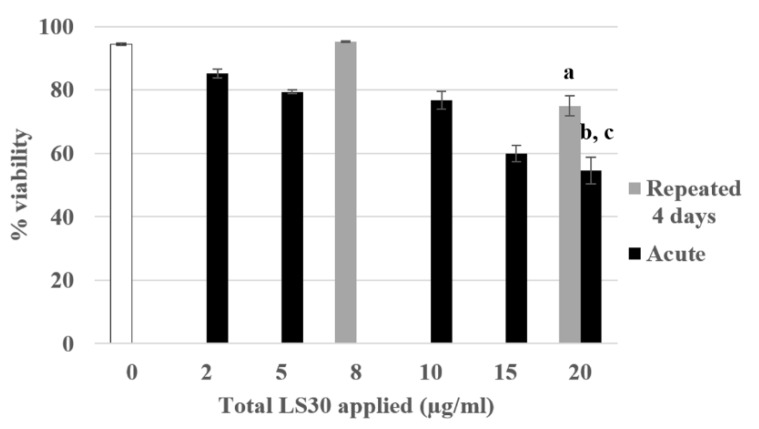
4-day chronic exposure of RAW264.7 to LS30 NP compared with acute exposure. Cell viability with propidium iodide by flow cytometry (a: repeated 5 µg/mL versus CTL, *p*-value < 0.001; b: acute 20 µg/mL versus CTL, *p*-value < 0.001; c: acute 20 µg/mL versus repeated 5 µg/mL, *p*-value < 0.01).

Following these experiments of 4-day exposure with the model fluorescent silica SiG, we transposed our work on silica nanoparticles used in industry. The colloidal Stöber LS30 silica has also a diameter of 30 nm, the process of fabrication is similar to that of the SiG, i.e., a wet chemistry synthetic process [26]. As previously, the lethal dose 20 had been determined, and corresponded to approximately 20 µg/mL (Figure 7). The same experiments following the same scenario were carried out. First, the viability assay showed that the cells repeatedly exposed to 5 µg/mL have a significant decrease of their viability of 20% compared with the control. The acutely exposed cells to 20 µg/mL showed a decrease of 40% compared with the control in these experiments. These two exposure conditions were also significantly different from each other (repeated 5 µg/mL versus acute 20 µg/mL).

**Figure 8:**
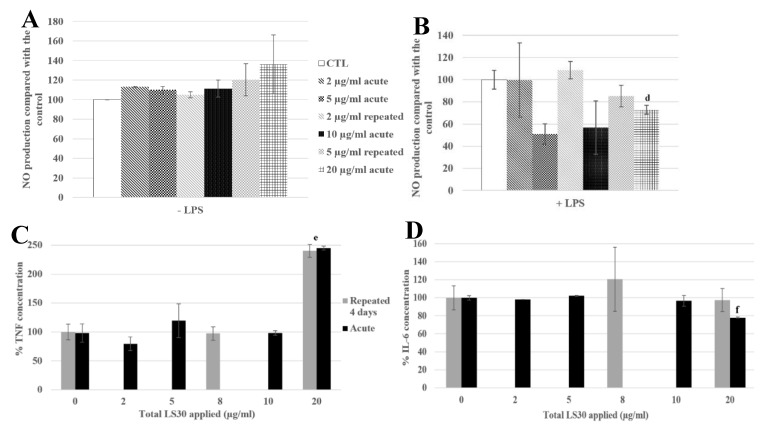
4-day chronic exposure of RAW264.7 to LS30 NP and 18h to LPS compared with silica acute exposure. (**A**,**B**) NO dosage with the Griess reagent by spectrophotometry without or with LPS stimulation (d: CTL versus acute 20 µg/mL, *p*-value < 0.01). (**C**) TNF releases of RAW264.7 cells exposed to LS30 only, measured by flow cytometry (e: repeated 5 µg/mL and acute 20 µg/mL versus CTL, *p*-value < 0.01). (**D**) IL-6 release measurement of LPS stimulated cells by flow cytometry (f: CTL versus acute 20 µg/mL, *p*-value < 0.01).

The NO production tests showed that in the absence of LPS stimulation, there were no significant differences between the control, the repeatedly exposed cells and the acutely exposed ones (Figure 8A). In the presence of LPS, the repeatedly exposed cells to 4 × 5 µg/mL and the control cells showed a similar NO production, whereas the acutely exposed cells to the LD20 showed a significant decrease of 27% compared with the control.

Regarding the cytokine releases, in the absence of LPS stimulation, the TNF secretion was 2.4 fold higher for 4 × 5 µg/mL and 1 × 20 µg/mL exposures than in the control (Figure 8C). The IL-6 secretion was decreased, with LPS stimulation, by the exposure to 20 µg/mL (22% compared with the control), and significantly different from the 4 × 5 µg/mL exposed cells, which were not different from the control cells.

#### 3.3.4. Physiological Situation of Co-Exposure: SiO_2_ and *E. coli*

**Figure 9:**
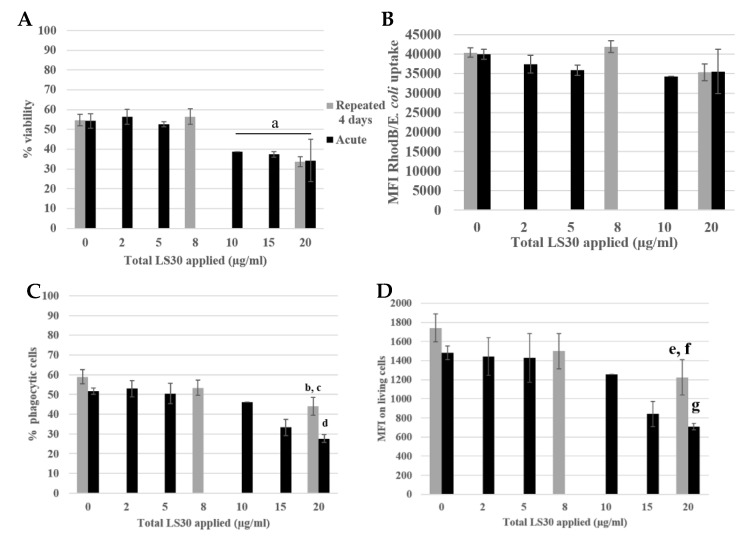
Phagocytosis assay of LS30 exposed cells, stimulated by Escherichia coli. (**A**) Cell viability (a: CTL versus acute 10 µg/mL, 15 µg/mL, acute 20 µg/mL and repeated 5 µg/mL, *p* value < 0.05). (**B**) *Escherichia coli* uptake followed by rhodamine B fluorescence. (**C**) Proportion of phagocytic cells (b: CTL versus repeated 5 µg/mL, *p*-value < 0.05; c: repeated 5 µg/mL versus acute 20 µg/mL, *p*-value < 0.01; d: CTL versus acute 20 µg/mL, *p*-value < 0.01). (**D**) Phagocytic activity of the cells (e: CTL versus repeated 5 µg/mL, *p*-value < 0.05; f: repeated 5 µg/mL versus acute 20 µg/mL, *p*-value < 0.01; g: CTL versus acute 20 µg/mL, *p*-value < 0.01.

**Figure 10:**
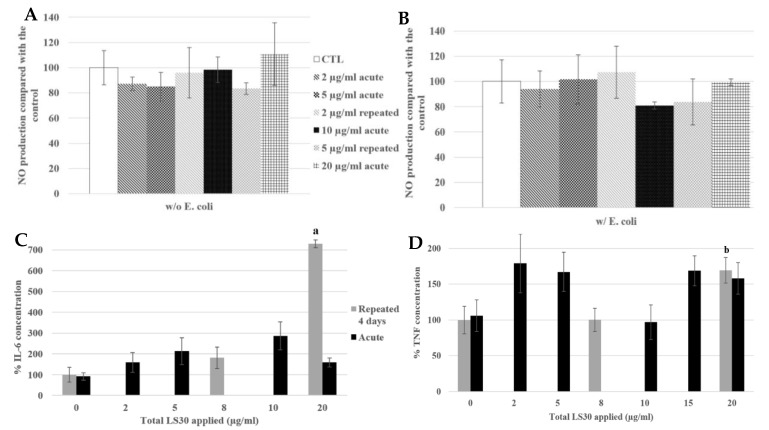
4-day chronic exposure of RAW264.7 to LS30 NP and 18 h to Escherichia coli compared with silica acute exposure. (**A**,**B**) NO dosage with the Griess reagent by spectrophotometry without or with *E. coli* stimulation. (**C**,**D**) Cytokine release measurement of LS30 exposed cells, in absence of *E. coli* stimulation by flow cytometry, respectively IL-6 and TNF. (a: CTL versus repeated 5 µg/mL, *p*-value < 0.01; b: CTL versus repeated 5 µg/mL, *p*-value < 0.05).

In all the previous experiments, the bacterial encounter that occurs in vivo was mimicked by an exposure to LPS. To further increase the relevance of our experiments, the repeated exposure to silica was combined to an exposure to killed bacteria. The cells were exposed for 4 days to the amorphous silica LS30, and for the last 18 h they were co-exposed to *E. coli* (which have been heat-killed after rhodamine B uptake) or LPS. Different doses were tested, and to be physiologically relevant with a signal measurable by flow cytometry, the bacterial concentration chosen was 30 bacteria per cell (b/c). This dose corresponded to a basal dose of bacteria in a non-sterile environment. For this co-exposure scenario, the viability and the bacteria fluorescence were measured, the phagocytosis, the nitric oxide (NO), and the cytokines were assayed. The results are presented in Figure 9 and Figure 10.

The viability is presented in Figure 9A, the LD20 was obtained for an acute 20 µg/mL dose and repeated 4 × 5 µg/mL). The *E. coli* uptake was measured by flow cytometry following the rhodamine B fluorescence. There were no differences between the different condition and the control. The proportion of phagocytic cells was decreased for the repeated 5 µg/mL compared with the control (12% decrease) and there was a decreased of 30% for the acutely exposed cells to 20 µg/mL compared with the control. Then, the phagocytic activity of the cells was measured. The activity of the repeatedly exposed cells to 4 × 5 µg/mL was 30% lower than the control, and the 1 × 20 µg/mL acutely exposed cells were 60% less active than the control.

We still obtained no intrinsic effect of LS30 in NO production whatever the LS30 exposure scenario. In presence of *E. coli* stimulation, the NO production was doubled in all the conditions (from about 7 µM in control without stimulation to 16 µM with *E. coli* stimulation).

In absence of stimulation, Il-6 release was increased 7.3-fold by the 4 × 5 µg/mL exposure compared with the control. The TNF was also increased by the repeated 5 µg/mL exposure, 1.7-fold compared with the control. The cytokine releases of IL-6 and TNF after *E. coli* stimulation were equivalent between all the conditions, there were no differences between the control, repeatedly exposed cells and the acutely ones. This suggested that the bacterium stimulation, which induced stronger effects than LPS alone, had erased the effects of LS30 exposure.

## 4. Discussion

In the context of an increasing use of amorphous silica in many products, it appears important to assess the effects of silica in a repeated exposure mode, and to compare the results to those obtained for the classical single dose exposure most often used in in vitro toxicology. As a target cell type, we used a macrophage cell line, as macrophages are the scavenger cells dealing with particulate materials in our body, and are also key players in inflammatory and immune responses. Then, two different exposure scenarios were performed to address these questions: a single, acute exposure and 4-day repeated exposure. Firstly, concerning silica internalization itself, it was shown that the SiO_2_ NP were internalized and accumulated in the cells, without any effect of the fluorescent dye. Furthermore, the intensities of fluorescence of the repeated doses were similar to the acute dose corresponding to the cumulative chronic dose. A major difference, however, is that repeated exposure did not produce the same effect on cellular viability than a single exposure to the same cumulated dose. For example, the acute 20 µg/mL has a decrease of 24% of the cell viability compared with the control, whereas the repeated exposure to even 4 × 10 µg/mL (40 µg/mL in a cumulative way) led to the same viability as the control. Similar observations were made with the LS30 silica concerning the signaling functions of macrophages: for 4-day exposure, the cumulative LD20 (4 × 5 µg/mL) applied in a daily treatment has an effect different from the LD20 in acute treatment. This, combined with the adhesion changes could modify the functionalities of the cells and their responses to SiO_2_ NP. One of the characteristic of the macrophages is the cell migration to the inflammation site. The acute SiO_2_ NP exposure did seem to modify the cell adhesion, which have been already see with ZrO_2_ NP, whereas ZnO_2_ NP induced a decrease of adherence ability of macrophages [27]. In the repeated exposure scenario, the apparently reduced adhesion was observed in control and exposed cells, so that it should be attributed to the long time at confluence rather than the exposure to silica.

For the second axis of the project regarding the cross-toxicity with bacteria, it was observed that the bacteria were internalized, regardless of the pre-exposure to silica (except the LD20 cumulative or acute doses) (data not shown). We used *Escherichia coli* as a bacterium stimulation because of it contained LPS, allowing a direct comparison with the classical LPS alone model stimulation. The effect of the LPS component and of the complete bacteria is reported in this study. At the bacterial concentration chosen (30 bacteria per cell), there was no effect on the cell viability in the absence of silica or for an acute exposure to silica at doses lower than the LD20 (i.e., 20 μg/mL for this cell line). This bacteria concentration corresponded to dose that the immune cells can encounter because of the non-sterile environment of our organism. The acute exposure to a high dose of silica and bacteria may have a synergistic effect on the cell viability.

Concerning one of the important functions of this cell type, namely phagocytosis, a decrease of phagocytic cells has been observed in both acute 20 µg/mL and repeated 5 µg/mL conditions, respectively, 64% and 38% compared with the control. However, the cells were more active to phagocytose the red beads in repeatedly exposed cells. Although the phagocytosis was decreased by the silica exposure, the phagocytic function was much less impacted by the repeated exposure than the acute one.

After these observations, the signaling function of macrophages was probed with the NO production and the cytokine release assays.

With the first model (RAW264.7 and SiG, stimulated or not with LPS), the NO production was modified. For the intrinsic effect of SiG, the chronic exposure resulted in a 15% decrease of the basal NO production, compared to unexposed cells, whereas the acute exposure led to an increase of 16%. Therefore, the silica nanoparticles have a proinflammatory effect in case of one high dose exposure but the intrinsic effect of repeated exposure decreases the NO production compared to the nonexposed cells. The opposite trend was observed with the LPS-stimulated cells, with a moderate, nonsignificant increase of 5% for the 4 × 5 µg/mL-chronic dose and a decrease of 30% for the 20 µg/mL-acute dose. The effect of exposure scenarios were opposed, with or without LPS stimulation. Then, the cytokine releases were also modified with silica exposure. In absence of LPS stimulation, there was an intrinsic effect of SiG, with an increase of TNF release in repeated and acute exposure, respectively, 3- and 5-fold compared with the control. With LPS stimulation, the exposure mode to silica have no significant effect on TNF release, but the IL-6 release was decreased, 80% for the repeated 5 µg/mL and 70% for the acute 20 µg/mL. The silica exposure altered the signaling response of macrophages in case of infection, and according to the silica exposure mode, the response was also different.

With the second model (LS30 with or without LPS), we did not observe the same effect. The LS30 did not modify the NO production, there was no intrinsic effect of the LS30 (in the absence of LPS). Indeed, with the LPS stimulation a decrease of the NO production had been observed in acute exposure to LD20. The co-exposure led to a synergistic effect on the NO production.

The co-exposure of RAW264.7 to 4 × 5 µg/mL LS30 silica and *E. coli* for 18 h did not affect the NO production, compared to the control, unlike the scenario 1 × 20 µg/mL LS30 and LPS. The *E. coli* stimulation seems to have erased the slight effect of the synergistic LS30/LPS effect, perhaps because of the higher NO response with complete bacterium stimulation.

The cytokine releases followed the same tendency for the exposure to LS30, with an increase of the proinflammatory cytokine secretion (TNF-α), in absence of stimulation. Furthermore, in repeated exposure to 5 μg/mL of LS30 silica, there was an increase of IL-6 release. These up-releases of proinflammatory cytokines may lead to an abnormal response of the immune system with a hyper stimulation of the immune cells. The TNF-α is involved in inflammation and regulation of immune cells, in addition, some links have been suggested between this cytokine and the IBD and COPD diseases [28,29]. These results are again supporting the importance of the repeated exposure, by illustrating the diversity of cellular responses between the different exposure scenarios.

With LPS stimulation, a decrease of IL-6 for the acutely exposed cells to 20 µg/mL had been observed. However, the *E. coli* stimulation did not show the same observation, as there was no effect of the silica exposure on the cytokine releases. These results, as those obtained for the NO production (with LPS or *E. coli*) shed light on the specificity of the stimulation source. The macrophage responses to LPS or complete bacteria were clearly different, even though the silica NP exposure was identical (LS30).

Herein, we observed that the inflammatory response of macrophages to silica was specific to the precipitated silica type (even though the physical parameters were similar), the exposure mode (acute or repeated) and the biological stimulus (bacterial LPS component or complete bacteria). This model is clearly relevant to study the long time effects of silica NP exposure, it would be a great alternative to in vivo models on rodents, which have been already used to investigate the chronic effects of amorphous silica [30]. An in vivo study of lung mice has also reported different responses between the single and the repeated inhalation exposure to ZnO_2_ NP [31], and also on silica where the absence of direct interference between nanosilica and bacterial clearance of macrophages has been observed [32]. In the context of minimization of animal experimentation, the development of in vitro systems is desirable, at least for the initial phases of the toxicological assessment of substances including nanomaterials. In this frame, advanced in vitro systems have been shown to be predictive for silver nanoparticles [33]. In addition, macrophage cell lines have brought interesting primary results, as exemplified on silver nanowires, where an advanced use of in vitro macrophage systems can recapitulate some of the events observed on in vivo mouse models [34]. In this respect, our in vitro results parallel those observed in vivo on the absence of effects of nanosilica on bacterial clearance by macrophages [32].

## 5. Conclusions

In conclusion, this work brought useful information about the silica NP. First, the SiO_2_ NPs are accumulated by the macrophages and change the adhesion properties of the cells. They interfere with the inflammation response to bacteria and modify the immune cell signalization by increasing the TNF-α and IL-6 releases. All these results lead to conclude that the acute exposure to low doses of NPs seems to not be toxic for the macrophages and the immune system. However, the repeated exposure to the same doses affects the cell viability, the phagocytosis and their signalization in case of the encounter with pathogens. This has to be taken into account to raise awareness the industrial workers to the risks at which they are exposed. It must be kept in mind that alveolar macrophages have a lifespan of several months.

## Figures and Tables

**Table 1:** Nano-size distribution of silica nanoparticles in water and culture media by DLS. LS30 for Ludox^®^ LS silica from Grace, SiG for Sicastar^®^-greenF, RPMI for Roswell Park Memorial Institute 1640 medium, FBS for fetal bovine serum. The Z-average of particles and their proportion in the solution are reported.

Nanoparticle	Solvant	Peak 1	Peak 2	Peak 3
SiG (40 µg/mL)	H_2_O	33.8 nm (100%)	0	0
	RPMI	34.4 nm (100%)	0	0
	RPMI 10% FBS	7.99 nm (22.8%)	43.5 m (74.4%)	527 nm (2.9%)
LS30 (20 µg/mL)	H_2_O	24 nm (100%)	0	0
	RPMI	22.6 nm (100%)	0	0
	RPMI 10% FBS	10.3 nm (17.7%)	63.3 nm (82.3%)	0
Media	H_2_O	0	0	0
	RPMI	0	0	0
	RPMI 10% FBS	39.7 nm (100%)	0	0

## Supplementary Materials

The following are available online at https://www.mdpi.com/2079-4991/10/2/215/s1, Table S1: Nano-size distribution of silica nanoparticles in water and culture media by DLS.
