# Supplementary material for: Repeated vs. Acute Exposure of RAW264.7 Mouse Macrophages to Silica Nanoparticles: A Bioaccumulation and Functional Change Study
Source: Nanomaterials (Basel). 2020 Jan 27;10(2):215. doi: 10.3390/nano10020215 (PMC7074975; doi:10.3390/nano10020215)
Supplement: Supplementary file 1 [file nanomaterials-10-00215-s001.pdf]

	SiG 40 µg/ml	LS30 20 µg/ml
**H_2O**	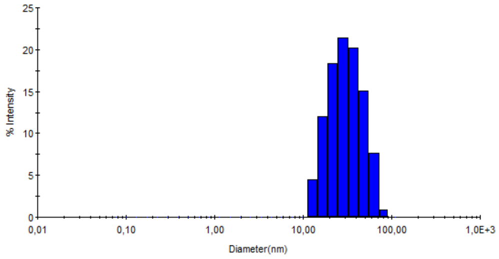	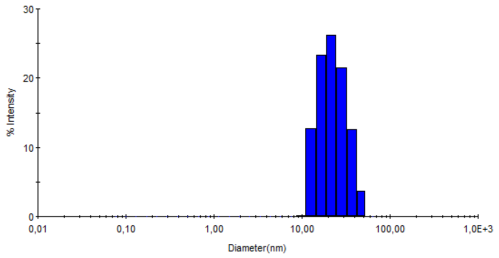
**RPMI**	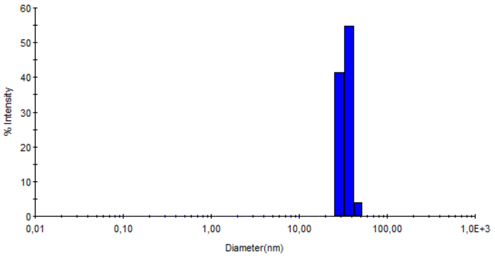	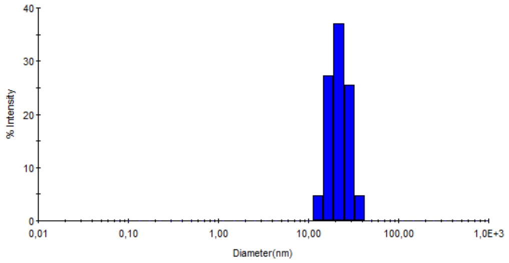
**RPMI 10% FBS**	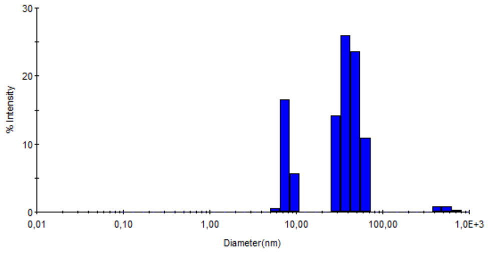	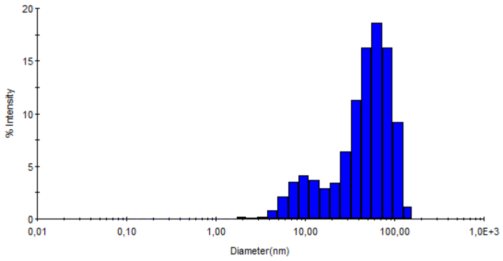

***Supplementary table S1*: Nano size distribution of silica nanoparticles in water and culture media by DLS.** *SiG for Sicastar®-greenF, LS30 for Ludox LS30, RPMI for Roswell Park Memorial Institute 1640 medium, FBS for fetal bovine serum. Distribution of intensity of nanoparticle population depending on the diameter, in water and culture media.*
